# DeepBand: A Deep Learning-Enabled Multi-Stage Pipeline for Continuous Automated Quantification of Lateral Flow Assays

**DOI:** 10.3390/diagnostics16182927

**Published:** 2026-09-10

**Authors:** Manan Vij, Alex J. Rai

**Affiliations:** Department of Pathology & Cell Biology, Columbia University Irving Medical Center, 622 West 168th Street, New York, NY 10032, USA

**Keywords:** lateral flow assay, artificial intelligence, convolutional neural network

## Abstract

**Background/Objectives:** Lateral flow assays (LFAs) are widely used point-of-care diagnostic devices due to their low cost, portability, and ease of use. However, most LFAs provide only qualitative results, limiting their utility for applications requiring continuous biomarker monitoring. This study introduces DeepBand, a deep learning-enabled multi-stage framework designed to automate the continuous quantification of analyte concentrations from unstandardized smartphone-captured lateral flow assay (LFA) images. **Methods:** A publicly available dataset containing 672 COVID-19 LFA images corresponding to four analyte concentrations (0.0, 1.8, 3.7, and 7.4 ng) was analyzed. A multi-stage pipeline was developed consisting of: (1) YOLOv11-based object detection to isolate the LFA cartridge from background artifacts, (2) a custom computer vision algorithm to identify and crop the test and control bands, and (3) a custom convolutional neural network (CNN) trained as a supervised regression model to predict continuous analyte concentrations. Data augmentation, hyperparameter optimization, and 5-fold cross-validation were used to improve model robustness. **Results:** The YOLOv11 model achieved approximately 99% mAP50 and 93.96% mAP95 for cartridge detection. Initial CNN models exhibited systematic underprediction of higher concentrations due to target imbalance; replacing mean squared error with Huber loss substantially improved performance, resulting in a final 20% held-out test set RMSE of 0.0292 ng. Analysis of HSV image channels demonstrated that the saturation-channel test-to-control intensity ratio was strongly correlated with analyte concentration (r = 0.94), consistent with the Beer–Lambert law governing LFA signal formation. Channel ablation studies confirmed the saturation channel as the most informative feature, while saliency mapping showed that the model primarily focused on biologically relevant test and control line regions. **Conclusions:** The proposed deep learning-enabled workflow, DeepBand, successfully integrates object detection, image processing, and CNN-based regression to provide automated quantitative interpretation of LFA results from smartphone images. Furthermore, the observed agreement between model behavior and Beer–Lambert theory suggests that the network learns biologically meaningful signal characteristics, supporting its potential for quantitative point-of-care diagnostics and longitudinal disease monitoring.

## 1. Introduction

Lateral flow assays (LFAs) are among the most widely used point-of-care diagnostic technologies due to their low cost, ease of use, rapid turnaround time, and compatibility with decentralized testing environments. These paper-based devices have become indispensable in clinical diagnostics, public health surveillance, and at-home testing applications, including infectious disease screening and pregnancy testing [1]. The widespread adoption of LFAs has highlighted their potential to support broader healthcare initiatives by enabling rapid diagnostic decision-making without requiring specialized laboratory infrastructure. However, despite their accessibility and convenience, most commercially available LFAs are primarily interpreted through visual inspection and provide qualitative or binary outcomes, such as positive or negative results. This limitation restricts their utility in applications where continuous monitoring of biomarker concentrations is required, including disease progression assessment, therapeutic monitoring, and post-treatment surveillance.

The wide availability of smartphones has enhanced the scope of low fidelity analysis (LFA) based diagnostics beyond just visual readouts. Smartphones offer high-resolution cameras, large processing power, and connectivity to allow not only imaging but also automated analysis and rapid transmission of results without the need for laboratory infrastructure. These attributes have made smartphones a preferred choice for alternate diagnostic platforms, which can offer both qualitative as well as quantitative information apart from enabling telemedicine and remote patient monitoring. Multiple reviews have emphasized the convergence of paper based microfluidics with smartphone based imaging and analysis as a paradigm for developing the next generation of point of care diagnostic devices [2,3,4]. In this regard it may be noted that smartphone based LFA quantification has progressed from simple pixel based intensity analysis to more involved computer vision and artificial intelligence based approaches which can improve accuracy, precision and ease of use while allowing for real world applications.

The proliferation of smartphones has made LFAs not only visual assays but also digital diagnostic devices that can perform quantitative measurements and remote healthcare applications. Initial studies showed that a smartphone camera is able to take and process colorimetric information from point-of-care assays as a low-cost and portable alternative to traditional laboratory readers without compromising clinical performance [5]. Later, cellphone-based reader devices have been engineered that unite the process of standardized picture taking, automatic image processing, and wireless communication, proving the viability of decentralized diagnostics and its real-time reports [6]. Reviews then revealed that smartphones are becoming an increasingly essential part of point-of-care diagnostics thanks to high-quality imaging, onboard computing, connectivity, and wide distribution, allowing the use of smartphones not only as a tool for image-based biosensing and quantitative analysis, but also for decision making by using cloud-based services and telemedicine [7]. All these advances have allowed the use of the latest achievements in computer vision and artificial intelligence algorithms for interpreting LFA pictures taken on smartphones and have made automated interpretation possible.

Recent advances in computer vision, machine learning (ML), and deep learning (DL) have created new opportunities to automate the interpretation of LFA results and reduce variability associated with human assessment. Early efforts focused on automated detection and classification of test bands using image-processing techniques and conventional machine learning methods. For example, Rashiku et al. developed an automated smartphone-based line detection system designed to improve sensitivity and reduce user-dependent interpretation errors [1]. More recently, large-scale machine learning systems have been developed to support real-world deployment of LFAs. The Automated Lateral Flow Analysis (ALFA) framework, developed as part of the United Kingdom REACT-2 study, utilized more than 500,000 user-submitted images to train models capable of classifying COVID-19 antibody tests with performance comparable to expert annotators and superior to untrained users, particularly for faint test lines [8]. Similarly, deep learning approaches have been applied to CRISPR-Cas-based LFAs, where Xue et al. demonstrated that convolutional neural networks could achieve 96.5% classification accuracy on smartphone-captured images without requiring controlled imaging conditions or specialized hardware [9]. These studies demonstrate the growing feasibility of deploying AI-assisted diagnostic systems in home and field settings.

Despite these advances, the majority of existing ML and DL approaches have framed LFA interpretation as a classification problem. Deep learning-assisted platforms such as SMARTAI-LFA have achieved impressive performance for identifying positive and negative test outcomes from smartphone images, reporting accuracies exceeding 98% across large clinical datasets [9]. Likewise, comparative studies have shown that convolutional neural networks often outperform traditional machine learning approaches when classifying noisy LFA images [10]. However, classification-based approaches fundamentally constrain outputs to a discrete set of categories, limiting their ability to capture the continuous nature of analyte concentrations encountered in many diagnostic applications. Consequently, while these systems can accurately determine whether a test is positive or negative, they provide limited information regarding biomarker burden or concentration-dependent changes over time.

Quantitative interpretation of LFAs presents a distinct challenge and remains comparatively underexplored. The theoretical basis for quantitative analysis originates from the Beer–Lambert law, which describes the relationship between analyte concentration and optical signal intensity. Within the assay’s linear dynamic range, increasing analyte concentration produces proportional increases in signal intensity, enabling concentration estimation from image-derived measurements. However, outside this range, signal saturation occurs, and the relationship becomes nonlinear, reducing quantitative reliability. The lower boundary of quantification is similarly constrained by the assay’s limit of detection, below which signal intensity cannot be reliably distinguished from background noise. Accurate quantification therefore requires both robust image analysis methods and an understanding of the biological and optical mechanisms governing signal formation.

Several studies have explored quantitative approaches for LFA. Zhang et al. developed a smartphone-based quantitative detection framework for multi-test-line LFAs using image-processing techniques combined with a support vector machine (SVM) for concentration estimation [11]. Although convolutional neural networks were incorporated during preprocessing, the final quantitative predictions relied on traditional machine learning methods rather than an end-to-end deep learning framework. More recently, Davis and Tomitaka demonstrated the feasibility of estimating analyte concentrations from smartphone-captured LFA images using machine learning techniques and established a publicly available dataset specifically designed for quantitative LFA [12]. These studies highlight the potential of image-based quantification but also reveal several remaining challenges, including robust handling of real-world image artifacts, automated localization of diagnostically relevant regions, and the need for models capable of directly predicting continuous analyte concentrations.

To address these limitations, there is growing interest in integrated deep learning workflows that combine automated image preprocessing with quantitative prediction. Such approaches are particularly attractive because they can reduce reliance on handcrafted feature engineering while improving scalability and robustness for point-of-care deployment. However, quantitative deep learning frameworks that incorporate automated region-of-interest detection, isolate biologically relevant signal regions, and perform continuous concentration estimation within a unified workflow remain relatively limited. Furthermore, few studies have investigated whether the features learned by these models correspond to the biological and optical mechanisms governing LFA signal generation. These gaps motivate the development of a multi-stage deep learning pipeline that not only performs quantitative concentration prediction but also enables examination of the relationship between model behavior and established principles such as the Beer–Lambert law.

In this study, we present DeepBand, a deep learning-enabled multi-stage workflow for the automated quantification of analyte concentrations from smartphone-captured LFA images. The proposed framework consists of three stages: (1) object detection using YOLOv11 to isolate the LFA cartridge from background artifacts, (2) custom image processing to identify and crop the test and control bands, and (3) a convolutional neural network trained as a supervised single-output regression model to predict continuous analyte concentrations. Using a publicly available COVID-19 LFA dataset containing 672 images across four concentration levels, we evaluate the feasibility of continuous concentration prediction rather than categorical classification. Beyond predictive performance, we investigate the biological basis of the model’s decision-making process through channel correlation analyses, channel ablation experiments, and saliency mapping. Our results demonstrate that the saturation component of the HSV color space exhibits a strong correlation with analyte concentration and serves as the most informative feature for prediction, consistent with the Beer–Lambert relationship governing LFA signal formation. Furthermore, saliency analyses reveal that the model primarily focuses on biologically relevant test and control line regions. Together, these findings demonstrate that quantitative LFA interpretation can be achieved through an integrated deep learning workflow while maintaining alignment with established biological principles, supporting future applications in point-of-care diagnostics and longitudinal biomarker monitoring.

## 2. Materials and Methods

Images used in this study were obtained from the publicly available COVID-19 lateral flow assay (LFA) dataset published by Davis and Tomitaka [12]. The dataset consists of 672 smartphone-captured images of LFAs corresponding to four analyte concentrations (0.0, 1.8, 3.7, and 7.4 ng). The objective of this study was to develop a deep learning-enabled framework capable of predicting analyte concentration directly from LFA images as a supervised regression task. The biological rationale for quantitative interpretation of LFAs is based on the Beer–Lambert law, which describes the relationship between analyte concentration and optical signal intensity. Within the assay’s linear dynamic range, signal intensity is approximately proportional to analyte concentration, enabling quantitative estimation from image data. At concentrations above this range, signal saturation occurs, and proportionality is lost, while concentrations below the limit of detection (LOD) become indistinguishable from background noise. The concentration values represented in the dataset were previously validated to lie within the assay’s linear operating range and within the assay LOD, ensuring that quantitative prediction is biologically meaningful [10].

To predict analyte concentrations from smartphone-acquired images, a three-stage workflow was developed. The workflow consisted of: (1) object detection and cartridge localization using YOLOv11, (2) custom image-processing algorithms to isolate the diagnostically relevant test and control bands, and (3) convolutional neural network (CNN)-based regression for analyte concentration prediction. This staged design was selected to progressively reduce irrelevant image content and ensure that only biologically meaningful signal regions were used during model training. By separating cartridge localization, signal extraction, and concentration prediction into distinct stages, the workflow was designed to improve robustness against image noise, lighting variability, and background artifacts commonly encountered in smartphone-captured images.

**Figure 1:**
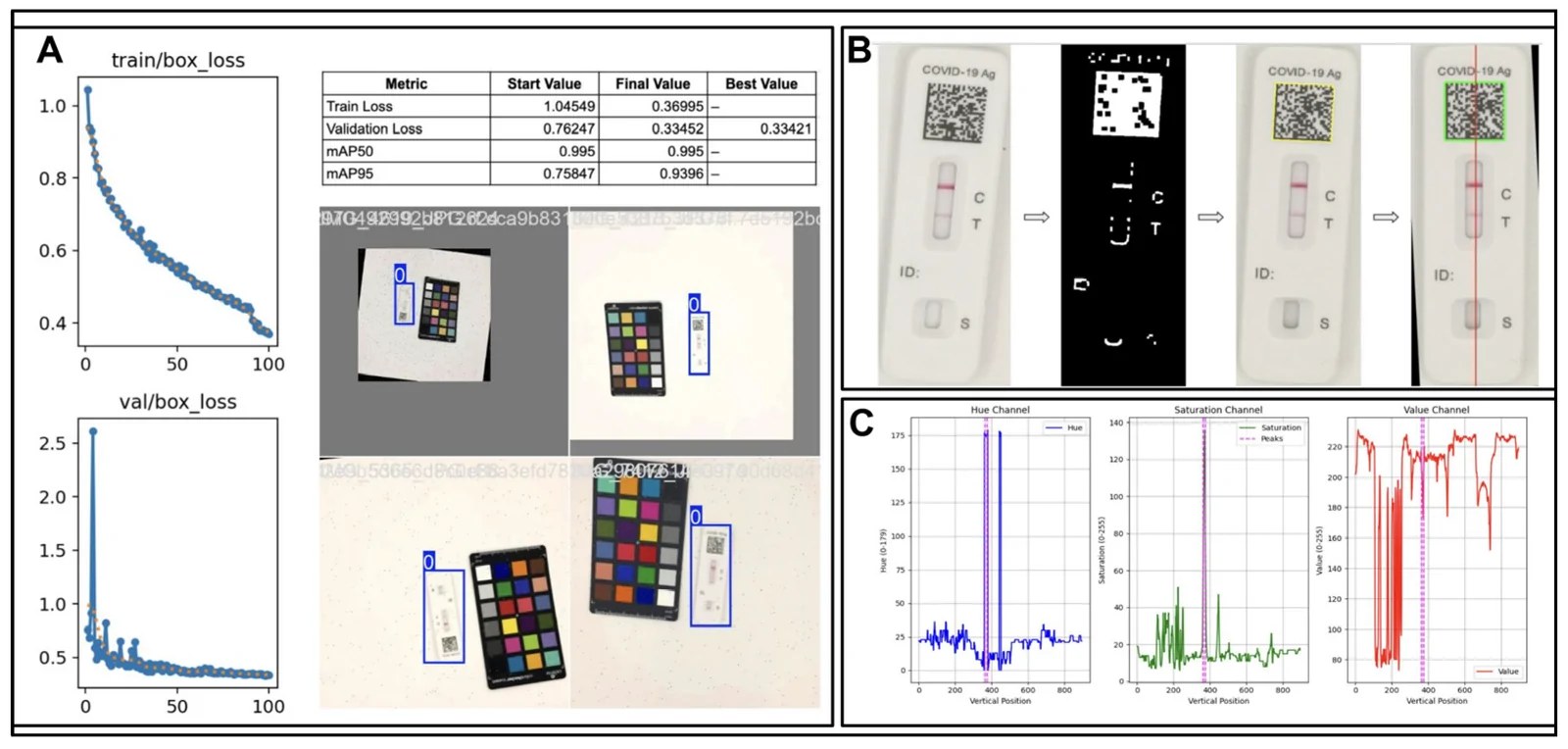
Multi-stage image processing and region-of-interest (ROI) isolation pipeline. (**A**) Raw smartphone image with the fine-tuned YOLOv11 bounding box prediction used to isolate the assay cartridge from background artifacts. (**B**) Schematic of the non-machine learning geometric workflow utilizing adaptive thresholding and QR-code orientation alignment. (**C**) Signal intensity profile along the central longitudinal axis across the hue, saturation, and value (HSV) channels, showing the localized control-line boundary and the optimal signal-to-noise ratio provided by the saturation channel prior to CNN input generation.

The first stage of the workflow involved automated detection of the LFA cartridge within the raw smartphone image. All 672 images were manually annotated with bounding boxes surrounding the cartridge region. Image augmentations were applied using the Roboflow platform (Des Moines, IA, USA), including random rotations (±10°), horizontal and vertical flips, Gaussian blur (up to 1 pixel), brightness adjustments (±10%), and random noise affecting approximately 1.5% of image pixels. These augmentations expanded the object-detection dataset approximately threefold. A pretrained YOLOv11 object-detection model was fine-tuned in Google Colaboratory using an 80:20 training-validation split. Model performance was evaluated using mean average precision (mAP) at intersection-over-union thresholds of 50% and 95%. Following training, the optimized model was applied to all original images to generate bounding box predictions. The detected cartridge regions were subsequently cropped and saved for further analysis, thereby removing external background artifacts and isolating the assay cartridge (Figure 1A).

Although cartridge detection removes extraneous background information, diagnostically relevant information is primarily contained within the test and control bands. Consequently, a second processing stage was developed to isolate these regions (Figure 1B). A custom image-processing pipeline was implemented using OpenCV (version 4.14.0) and did not require machine learning or deep learning methods. Images were first converted to grayscale and smoothed using Gaussian filtering before adaptive thresholding was applied to generate binary masks. Morphological operations were subsequently used to enhance structural features and facilitate contour detection. The cartridge QR code was identified through contour analysis and geometric filtering of approximately square-shaped regions. The QR code location was then used to estimate image orientation and calculate a central longitudinal axis passing through both the test and control bands (Figure 1B). To identify the control-band location, a custom peak-detection algorithm was applied to the hue (H), saturation (S), and value (V) channels of the image. Peak positions were calculated along the central axis, and the saturation channel consistently produced the most distinct control-line signal (Figure 1C). Following localization of the control band, fixed positional offsets were used to crop the image to include only the test and control regions. These HSV-space cropped images were saved and used as input for CNN training.

The final stage of the workflow involved a custom convolutional neural network designed to predict continuous analyte concentrations from the isolated test and control bands. Quantitative concentration prediction was formulated as a supervised single-output regression task. Images produced by the preprocessing pipeline were randomly divided into training (80%) and testing (20%) datasets. To improve model robustness and reduce overfitting, the training dataset was augmented tenfold, whereas the testing dataset remained unmodified. Image pixel values and concentration labels were normalized to a range between 0 and 1 using min-max normalization prior to training. Predicted values were converted back to the original concentration scale for evaluation and interpretation.

**Figure 2:**
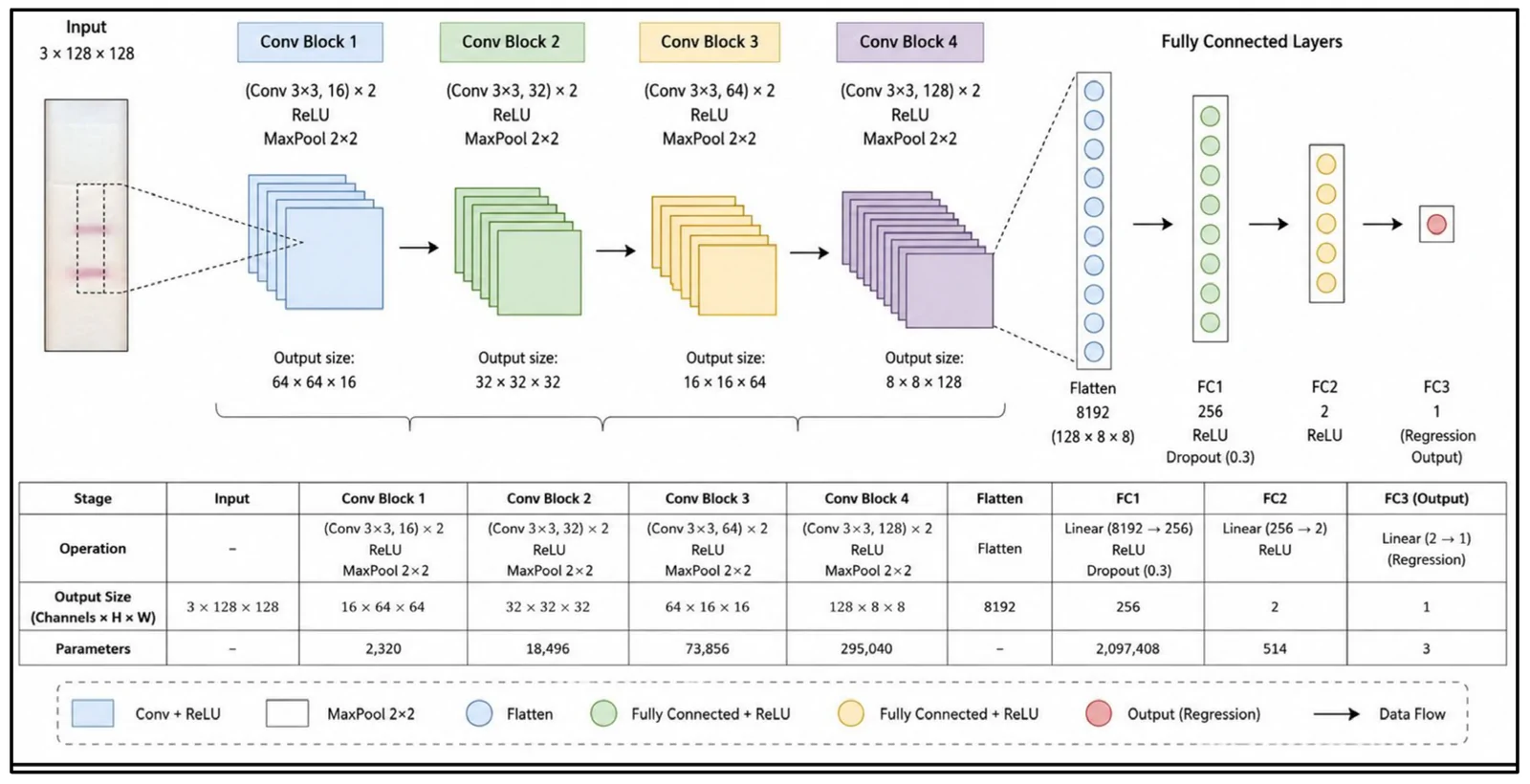
Schematic representation of custom CNN model used for LFA quantitation.

The CNN architecture consisted of four convolutional blocks, each containing two convolutional layers followed by rectified linear unit (ReLU) activation functions and max-pooling operations (Figure 2). Convolutional layers utilized 3 × 3 kernels with stride 1 and padding 1, while max-pooling layers employed 2 × 2 kernels with stride 2, progressively reducing image dimensions while increasing feature abstraction. Following feature extraction, the output feature maps were flattened and passed through fully connected layers. A dense layer containing 256 neurons was followed by ReLU activation and dropout (0.3 probability) to improve generalization. A second dense layer further reduced the feature representation before a final single-neuron output layer produced the predicted analyte concentration. Model optimization was performed using gradient-based backpropagation on an NVIDIA T4 GPU within Google Colaboratory. Initial models were trained using mean squared error (MSE) loss; however, following preliminary experiments, Huber loss was adopted as the final loss function due to improved performance across the full concentration range. Hyperparameter optimization was performed using grid search combined with 5-fold cross-validation, and all reported performance metrics were obtained using the optimized model configuration.

Image preprocessing and computer vision operations were implemented using OpenCV. Deep learning models were developed in Python (version 3.13.15) using the PyTorch (version 2.11.0) framework, and YOLOv11 object-detection models were trained using the Ultralytics (version 8.4.137) implementation. This study used a publicly available, de-identified dataset and did not involve human subjects, animal experiments, or collection of identifiable patient information; therefore, institutional review board approval was not required. The dataset used in this study is publicly available through the publication by Davis and Tomitaka [12]. Figure 2 was generated using ChatGPT (version GPT-5.6 Luna).

## 3. Results

### 3.1. Model Optimization and Loss Function Evolution

**Figure 3:**
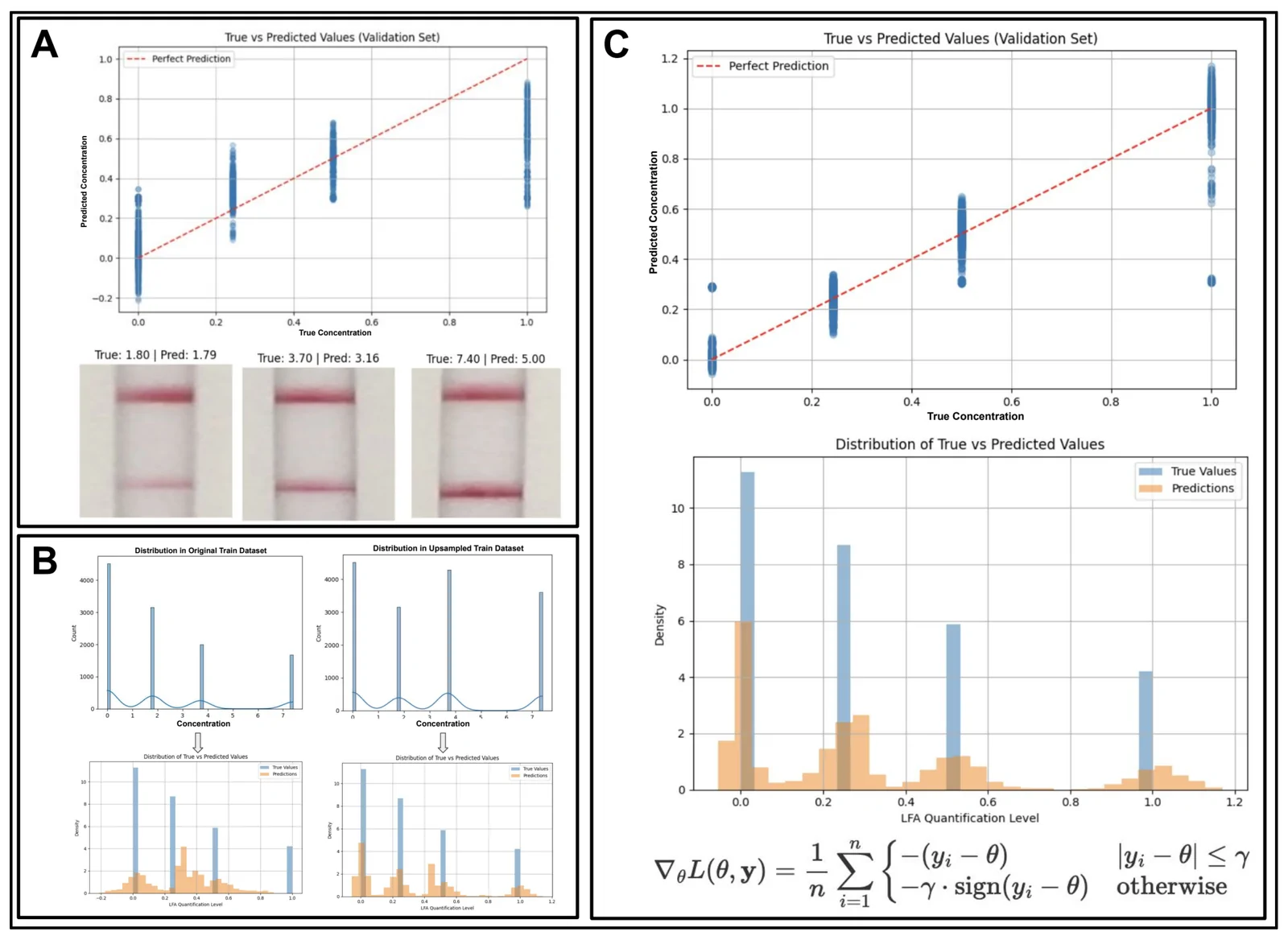
Algorithmic mitigation of dataset imbalance using loss function optimization. (**A**) Distribution of the original right-skewed training dataset alongside the corresponding baseline model predictions, demonstrating a systematic under-prediction bias at elevated concentration levels due to data scarcity. (**B**) Model predictions following targeted data upsampling of the high-concentration cohorts, demonstrating restored predictive accuracy through manual dataset balancing. (**C**) Optimized continuous model predictions across the full analytical range achieved by transitioning the optimization objective from standard mean squared error (MSE) loss to a robust Huber loss function without modifying the original data structure.

The initial model exhibited a progressive decrease in training loss but demonstrated significant predictive limitations during validation on higher-concentration test cases. For instance, while the model accurately predicted baseline concentration, it demonstrated notable inaccuracy at elevated levels (Figure 3A). To identify the underlying cause of this systematic bias, an analysis of the training dataset’s distribution was conducted. The baseline training set exhibited a severe right-skewed distribution, where elevated target concentrations were significantly under-represented in the dataset. This data scarcity directly correlated with the model’s failure to capture higher-end values. While targeted data upsampling (increasing data augmentation factors strictly on the 3.7 ng and 7.4 ng cohorts) successfully balanced the distribution and restored predictive accuracy at the high end, an alternative algorithmic solution was pursued to maintain the integrity of the original dataset structure without tampering with the original data (Figure 3B).

Consequently, the optimization objective was shifted from a standard mean squared error (MSE) loss function to a Huber loss function. Under MSE optimization, frequent low-concentration examples dominate the gradient update direction, allowing the network to settle into a locally optimal parameter set that minimizes global error while accepting high-error penalties on rare, high-concentration samples. Huber loss mitigates this by transitioning from a quadratic penalty for small residuals to a linear penalty for large residuals. This modification prevents the rare high-concentration samples from either destabilizing gradient descent or being ignored in aggregate. As a result, the model optimized evenly across the entire target range without requiring artificial dataset modifications (Figure 3C). The final optimized architecture was evaluated using a 5-fold cross-validation grid search to select optimal learning rates and optimizers, achieving a highly stable final average training loss of 9.48 × 10^−5^ across all folds (Table 1).

### 3.2. Color Space Selection and Channel Ablation

To mitigate the impact of environmental lighting variations, exposure settings, and shadows common in unstandardized images, the input dataset was converted entirely to the HSV color space prior to neural network training. Preliminary exploratory testing using standard RGB images yielded exceedingly poor quantification results, validating the necessity of an HSV-centered pipeline. A peak-detection algorithm applied directly to the HSV space demonstrated superior performance in consistently locating control and test line positions to generate the localized model inputs.

**Figure 4:**
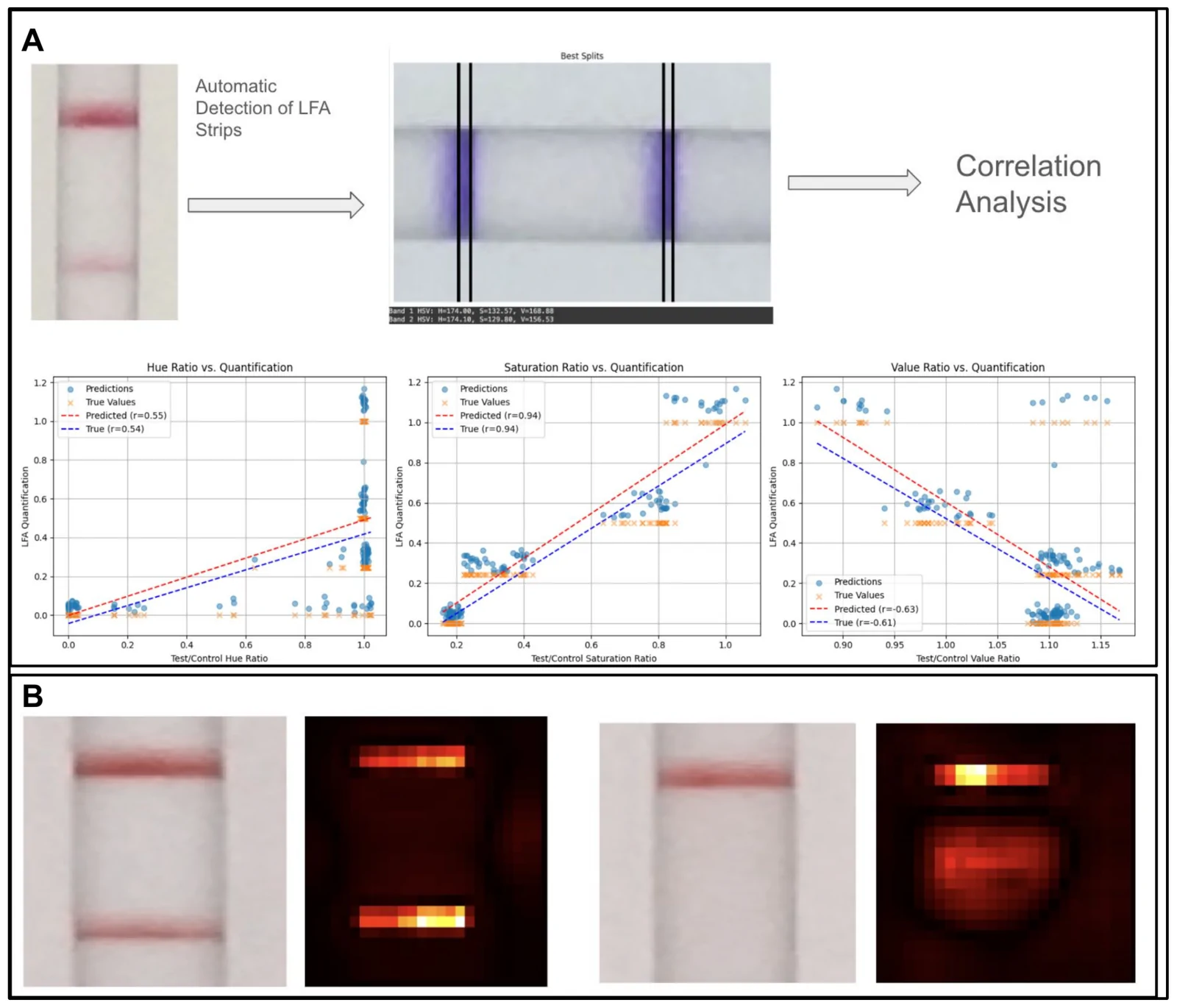
Feature attribution, color space validation, and network interpretability analysis. (**A**) Linear regression curves modeling the relationship between true/predicted lateral flow assay (LFA) analyte concentrations and the test/control intensity ratios across individual hue, saturation, and value (HSV) channels, validating the biological alignment of the saturation channel with Beer-Lambert’s law. (**B**) Representative patch-occlusion saliency maps highlighting the model’s precise spatial focus on the biological test and control bands, showcasing a concentrated focus on true-positive strips versus a diffuse, expanded visual search pattern across the control region on true-negative (0.0 ng) strips.

To validate the biological relevance of the HSV channels, a non-machine learning geometric algorithm was implemented to establish tight boundaries around the test (T) and control (C) lines by maximizing the mean saturation between localized coordinate splits. The ratio of the average intensities (Test Intensity/Control Intensity) within a 10-pixel vertical region centered on the split axes was calculated across the H, S, and V channels. Utilizing a ratio rather than raw pixel intensities is critical to normalize variations introduced by design-specific parameters (e.g., microfluidic flow rates) or unstandardized imaging environments (e.g., varied ambient lighting). Linear regression analysis evaluating the correlation between these channel ratios and the true LFA concentrations revealed that the saturation (S) channel is exceptionally highly correlated with analyte concentration (r = 0.94), whereas the hue (r = 0.54) and value (r = −0.61) channels demonstrated moderate correlations. Plotting the CNN model’s predictions against these ratios confirmed that the network successfully learned this underlying analytical relationship (Figure 4A).

The strong coefficient correlation (r = 0.94) observed between the signal/test intensity ratio and the concentration prompts consideration of a simpler linear model. Linear regression resulted in an RMSE of 0.1095 ng (R^2^ = 0.9087) on a 20% held-out test set, compared with the 0.0292 RMSE of the CNN (R^2^ = 0.9998).

To rigorously quantify individual channel importance within the deep learning model, a channel ablation study was executed by sequentially masking each channel (setting pixel values to 0) during inference. While the ablation study confirmed that the saturation channel provides the most critical discriminative features due to its ability to capture color purity independent of brightness, the significant error spikes observed when omitting Hue and Value support the deployment of a fully integrated three-channel HSV training approach over a single-channel alternative (Table 2).

### 3.3. Interpretability via Saliency Mapping and Variance Reduction

To verify that the model was utilizing clinically relevant features rather than background artifacts, feature attribution maps were generated using a patch occlusion methodology. Small spatial patches were systematically masked with mean pixel values to measure corresponding drops in regression accuracy. The resulting saliency maps demonstrated that areas corresponding strictly to the LFA test and control bands caused the most severe performance degradation when occluded, confirming the model’s spatial focus on the biological regions of interest. Notably, for true-negative (0.0 ng) concentration strips, the saliency maps revealed an expanded, diffuse attention pattern surrounding the control line region. This computationally mirrors human visual inspection, where an observer expands their field of search across the expected region of interest to confidently verify the complete absence of a faint test line (Figure 4B).

## 4. Discussion

The fundamental paradigm of the DeepBand framework centers on a continuous regression pipeline capable of mapping unstandardized, open-source visual features directly to precise analyte concentrations [12,13]. This approach departs from traditional automated lateral flow assay (LFA) workflows, which typically rely on classification models to deliver binary or coarse, discrete diagnostic categories [10]. While classification suffices for acute, threshold-based screening, it is inadequate for chronic disease management, progressive monitoring, or early recurrence detection where subtle biomarker fluctuations carry significant clinical weight.

Operating a regression model on a skewed, open-source dataset, however, presented distinct optimization challenges. Under standard mean squared error (MSE) loss, frequent low-concentration samples dominated gradient updates, causing the model to prioritize local optimization at the lower range while accepting high-error penalties on rare, high-concentration samples. By shifting to a Huber loss function, the quadratic penalty for minor residuals was combined with a linear penalty for substantial deviations [13]. This prevented rare high-concentration gradients from being ignored or destabilizing the network, enabling stable continuous quantification across the entire target range without structural modification of the underlying dataset [13]. This robust convergence is supported by the 5-fold cross-validation grid search, which yielded a remarkably low RMSE of 0.0292 ng on a held-out 20% test set, proving that a software-only regression approach can reliably handle decentralized data imbalances.

A key innovation driving this performance is the integrated, multi-stage nature of the diagnostic pipeline. Rather than feeding raw, unmanipulated smartphone images directly into a neural network—which often leads to severe overfitting on background artifacts—the proposed architecture sequentially decouples localization from quantification [14]. The first stage leverages localized object detection to isolate the LFA cartridge from surrounding environmental noise. This is followed by automated cropping and geometric region-of-interest extraction, which isolate the test and control bands before passing the standardized matrices into the convolutional regression network. This multi-stage orchestration ensures that the deep learning model allocates its computational capacity entirely to biologically relevant features, serving as an architectural safeguard against the variations in perspective, distance, and orientation common in crowdsourced imagery [14].

Ensuring that the network learns true biological signals rather than exploiting background artifacts or image noise is critical for digital health informatics. Converting raw inputs to the HSV color space proved essential to decouple analytical data from ambient lighting variations and shadow distortions common in unstandardized web photos [14,15]. The linear regression analysis structurally validated this color space selection, revealing a powerful correlation (r = 0.94) strictly between the saturation channel ratio (Test/Control) and true LFA concentrations, contrasting sharply with the weak or inverse correlations shown by hue (r = 0.54) and value (r = −0.61). This high correlation provides a solid physical foundation grounded in Beer–Lambert’s Law, which dictates a linear relationship between a substance’s concentration and its optical absorbance. Because the physical accumulation of colloidal gold or fluorescent nanoparticle conjugates on an LFA strip enhances color purity rather than changing the fundamental color wavelength, the saturation channel functions as a clean digital capture of chemical absorbance [15]. By calculating the ratio of the test line saturation relative to the control line, the algorithm mathematically isolates this chemical signal and filters out environmental exposure changes [15]. The Value channel is inherently dominated by ambient lighting noise, while the Hue channel remains static since the underlying wavelength of the chemical conjugate does not shift. Although the baseline linear model based on the saturation channel test-to-control line ratio offers the advantage of straightforward interpretability, it substantially underperformed the CNN model, exhibiting an RMSE that was approximately five times higher. This result indicates that the simple ratio-based representation is insufficient to capture the complex visual information present in the images, whereas the CNN is able to learn richer spatial and contextual features that translate into significantly improved predictive performance.

The channel ablation study confirmed that the model’s internal representations align with this physical reality: masking the saturation channel triggered the highest inflation in prediction error (5088.0), confirming it as the primary carrier of discriminative information. Concurrently, the significant error inflation observed when omitting hue (3632.1) and value (2225.0) suggests that the network does not rely on a single channel in isolation; instead, it leverages the full HSV spectrum to contextually isolate the region of interest and algorithmically suppress ambient illumination artifacts [15]. The channel ablation study was conducted by zeroing individual input channels while keeping all other inputs unchanged. This perturbation may produce out-of-distribution inputs relative to the training data, potentially inflating the observed error increases. Consequently, the results are intended to provide a qualitative assessment of the model’s sensitivity to each channel and should not be interpreted as a rigorous attribution of feature importance.

Furthermore, feature attribution via patch-occlusion saliency mapping provided visual proof of the model’s focus. The highest importance metrics mapped precisely to the physical coordinates of the test and control bands rather than the plastic cartridge perimeter. The discovery of a more diffuse attention pattern surrounding the expected test line region, specifically within true-negative strips, is particularly compelling. It demonstrates that in the absence of an explicit signal, the network expands its field of search across the entire localized region of interest to verify a true negative—computationally mirroring the investigative visual scanning behavior of a human clinician.

Traditional automated LFA quantification approaches heavily depend on dedicated desktop optical readers or specialized smartphone hardware attachments, such as 3D-printed lightboxes, diffusers, or rigid calibration cradles [14,16,17]. While these physical attachments fix distance and normalize illumination, they introduce substantial manufacturing overhead, increase per-test costs, and create friction for rapid, decentralized public health distribution [18]. The software-centric framework presented here shifts the stabilization burden entirely from the physical environment to an automated algorithmic pipeline, enabling raw, smartphone-captured imagery to achieve comparable analytical utility [11,14].

A recognized limitation of the current regression framework, however, is the increased predictive variance observed at elevated concentration ranges, which may reduce sensitivity when distinguishing between closely spaced high biomarker concentrations. This behavior may be associated with the nonlinear response characteristics and signal saturation effects commonly observed in lateral flow assays at higher analyte levels. Additionally, although the model provides continuous concentration predictions within the evaluated range, the current study was validated using a single publicly available dataset containing four experimentally measured concentration levels. Future work should evaluate the generalizability of the proposed workflow using independent datasets, different LFA formats, broader concentration ranges, and more diverse imaging conditions.

**Figure 5:**
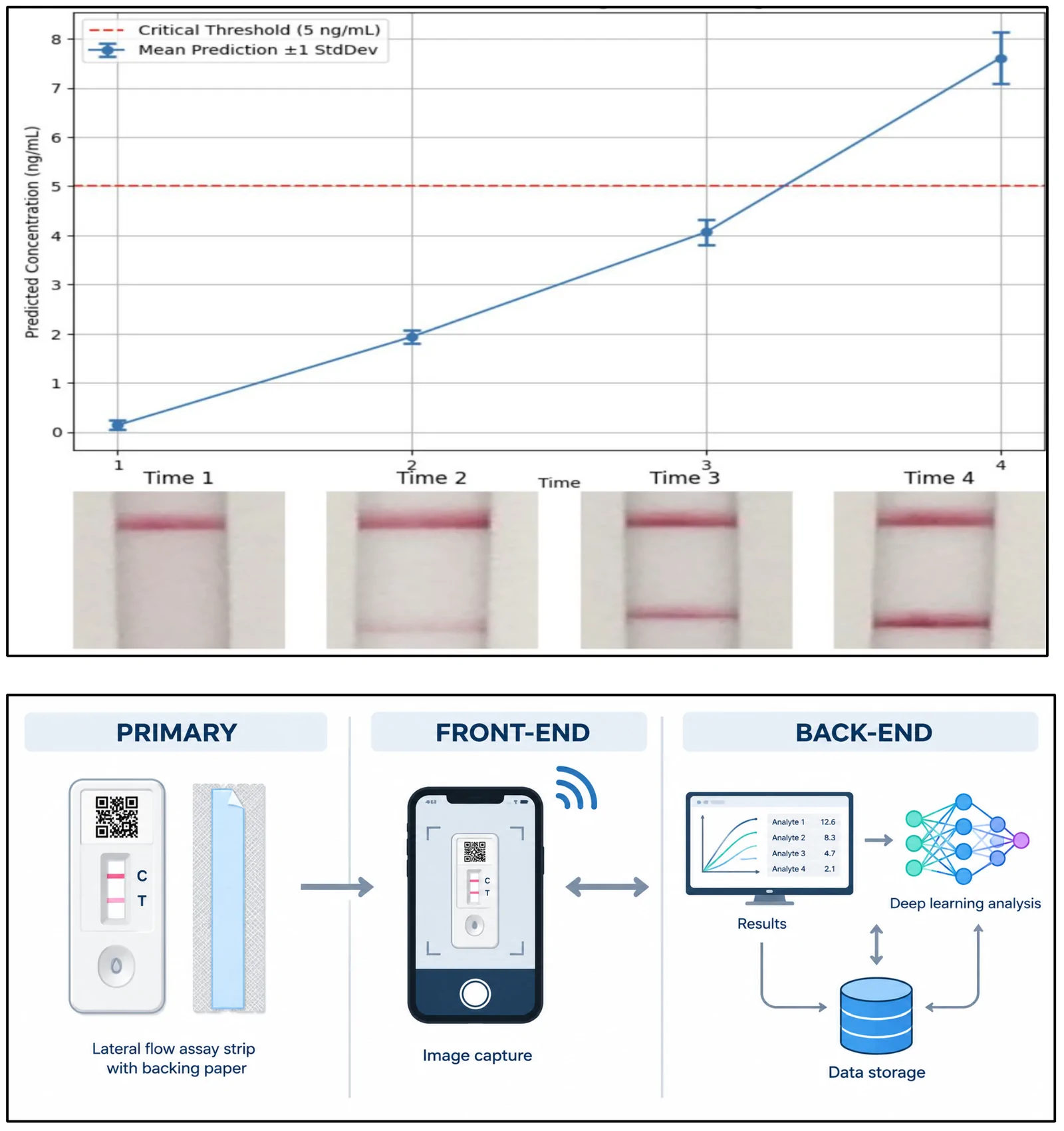
Simulated longitudinal clinical monitoring framework (**top** panel) and cloud ecosystem architecture (**bottom** panel). The schematic layout details the end-to-end smartphone-to-cloud data flow, illustrating user-end image capture on a standardized backing calibration card, backend region-of-interest (ROI) segmentation and HSV transformation, continuous convolutional neural network (CNN) regression quantification, and real-time longitudinal trend tracking for proactive clinical intervention.

To demonstrate the clinical utility of the developed quantification pipeline, a longitudinal monitoring framework was simulated (Figure 5). In post-treatment clinical environments—such as monitoring patients for disease recurrence—the framework supports remote, patient-driven tracking [18]. As this proof-of-concept figure demonstrates, patients can capture periodic images of diagnostic test strips at home, which are then algorithmically parsed to chart biomarker concentrations over time [16]. If a tracking sequence exhibits a rising trajectory that breaches a predefined clinical threshold, an automated alert can be triggered for physician intervention. To operationalize this pipeline, a complete smartphone-to-cloud ecosystem architecture was designed [18]. The workflow initiates via a mobile application interface where a user captures an LFA image. To reduce environmental perspective and distance errors, the strip is positioned on a standardized backing calibration card. The front-end mobile application uploads the raw image to a cloud-based backend server [18]. The server processes the file through successive stages: automated object detection, cartridge localization, region-of-interest cropping, HSV transformation, and CNN-based regression quantification. The quantified analyte concentration is returned to the user interface in real time while concurrently archiving the data to a secure database to facilitate longitudinal healthcare tracking and integration into Electronic Health Records (EHR) [18]. To transition this framework from a simulated proof-of-concept to an active clinical asset, immediate future work will focus on prospective clinical validation. The cloud-based backend will be deployed to evaluate active patient cohorts, utilizing real-world clinical datasets targeting specific disease biomarkers. This clinical scaling will evaluate the pipeline’s operational sensitivity and specificity under diverse user-end environments, establishing a validated blueprint for open-data-driven, decentralized digital diagnostics.

## Figures and Tables

**Table 1:** Hyperparameter optimization and performance evaluation of the continuous regression model via grid search and 5-fold cross-validation. The final validation loss is reported for each hyperparameter configuration across individual validation folds, with the top-performing configuration tracking the lowest average validation loss (lr = learning rate).

Batch Size	lr	Optimizer	Fold 1	Fold 2	Fold 3	Fold 4	Fold 5	Avg Final Val Loss
32	0.0005	Adam	6.73 × 10^−5^	8.85 × 10^−5^	9.35 × 10^−5^	1.29 × 10^−4^	9.54 × 10^−5^	9.48 × 10^−5^
32	0.0001	Adam	1.53 × 10^−4^	1.65 × 10^−4^	1.67 × 10^−4^	1.57 × 10^−4^	2.37 × 10^−4^	1.76 × 10^−4^
64	0.0005	Adam	0.00020	0.00020	0.00010	0.00020	0.00020	0.00018
32	0.001	Adam	8.18 × 10^−5^	6.99 × 10^−5^	4.94 × 10^−5^	9.05 × 10^−4^	3.54 × 10^−5^	2.22 × 10^−4^
64	0.001	Adam	0.00216	0.0057	0.00010	0.0044	0.0001	0.00206
32	0.001	SGD	0.0527	0.0523	0.0542	0.0516	0.0561	0.0534
32	0.0001	SGD	0.0527	0.0523	0.0542	0.0516	0.0561	0.0534
32	0.0001	SGD	0.0527	0.0523	0.0542	0.0516	0.0561	0.0534
64	0.001	SGD	0.0527	0.0523	0.0542	0.0516	0.0561	0.0534
64	0.0005	SGD	0.0528	0.0523	0.0542	0.0517	0.0561	0.0534
64	0.0001	SGD	0.0532	0.0529	0.0544	0.0516	0.0565	0.0537
64	0.0001	Adam	0.0539	0.0538	0.0551	0.0521	0.0573	0.0544

**Table 2:** Performance degradation and error impact metrics from the single-channel ablation study during model inference. Percent increases in prediction error are listed for the systematic masking of the individual Hue, Saturation, and Value (HSV) channels, quantifying the relative feature importance of each channel within the integrated deep learning regression pipeline.

Channel	Loss (Mean)	Loss (Std)	% Increase
Baseline	0.002	0.0002	
Hue	0.0739	0.023	3632.10%
Saturation	0.1027	0.0101	5088.00%
Value	0.046	0.0049	2225.00%

## Data Availability

The original contributions presented in this study are included in the article. Further inquiries can be directed to the corresponding author.
